# Prostate stereotactic body radiotherapy: quantifying intra-fraction motion and calculating margins using the new BIR geometric uncertainties in daily online IGRT recommendations

**DOI:** 10.1259/bjr.20220852

**Published:** 2023-04-22

**Authors:** Joseph M McNeice, Nandu Sanilkumar, Sophie E Alexander, James Talbot, Alison C Tree, Helen A McNair

**Affiliations:** 1 The Royal Marsden NHS Foundation Trust, London, UK; 2 The Institute of Cancer Research, London, UK

## Abstract

**Objectives:**

To measure the magnitude of intra-fraction prostate motion (IFPM) during stereotactic radiotherapy (SBRT) delivered without intra-fraction tracking.

To assess if current margins adequately cover IFPM.

To derive margins using new guidelines.

**Methods:**

IFPM was determined in 20 patients receiving 36.25 Gy in 5 fractions using 97 pairs of pre- and post-treatment cone beam CT (CBCT) scans. Correlation of time between CBCT acquisitions and motion was determined. The magnitude of IFPM was compared to current margins (6 mm isotropic, 3 mm posterior). Margins were calculated using IFPM alone and updated guidelines.

**Results:**

The averaged 3D root mean square IFPM was 2.5 mm (4.2 mm). Independent prostate motion was predominantly posterior (70%) and inferior (63%). There was weak correlation between posterior (ρ = 0.38) and inferior (ρ = 0.36) IFPM and time. IFPM greater than current margins occurred in 8 of 97 fractions, six in the posterior direction. Margins were ≤3.5 mm using IFPM alone and ≤3.3 mm Left 3.5 mm Right, 7.0 mm inferior, 3.7 mm superior, 4.4 mm anterior and 3.3 mm posterior using new guidelines, compensating for motion in 92% of fractions.

**Conclusions:**

Our current SBRT margins account for 92% of IFPM, predominantly posterior and inferior. Although updated guidelines suggest an increase in margins inferiorly, any increase must be balanced against the possibility of increased toxicity, particularly if biochemical control and side-effects are favourable with current practice.

**Advances in knowledge:**

The difference between current clinical margins and those determined using updated guidance is demonstrated. The implications must be considered against clinical outcomes.

## Introduction

Prostate cancer is the most common male cancer in the UK,^1^ with approximately 30% of all prostate cancer patients receiving external beam radiotherapy (EBRT).^2^ Techniques such as intensity modulated radiotherapy and image-guided radiotherapy (IGRT) have enabled dose escalation, hypofractionation and improved outcomes^3^ by taking advantage of the low α/β ratio of prostate cancer.^4,5^Hypo-fractionated radiotherapy has become the standard of care following the results of the CHHiP trial.^6^ The implementation of stereotactic radiotherapy (SBRT) has the potential to further reduce radiotherapy fractions, increase fractional dose to the tumour and minimise normal tissue irradiation.

The COVID-19 pandemic escalated the use of prostate SBRT as the National Institute for Health and Care Excellence COVID guidelines recommended centres “use the shortest safe form of treatment”, where treatment was unavoidable.^7^ Whether SBRT should be standard of care for prostate patients remains a matter of debate,^8,9^ however recently published data from the HYPO-RT-PC (ISRCTN45905321) and PACE-B (ISRCTN17627211) trials, comparing SBRT with conventional radiotherapy present comparable or slightly increased acute toxicity with similar long-term side effects.^10,11^ A large, pooled data analysis^12^and systematic review^13^ have shown comparable outcomes in survival and toxicity between SBRT and conventional treatments.

Safe delivery of SBRT demands precise treatment delivery. Online IGRT reduces set-up error, but residual errors remain, of which intra-fraction motion is a component. The impact of intra-fraction motion is potentially greater in SBRTtreatments due to longer treatment delivery time,withfewer fractions to average out motion. To ensure planning target volume (PTV) margins adequately compensate for such motion, audit is recommended when introducing SBRT.^14^ SBRT prostatemargins range from 2 mm to 5 mm^11,15,16^ with some utilising reduced margins in the posterior direction.^17,18^ Few studies have described margin derivation.^19^

Recommendations for evaluating the contribution of intra-fraction motion in online IGRT have recently been published.^20^ This study aims to evaluate intra-fraction motion when delivering prostate cancer SBRT, and calculate appropriate margins based on intra fraction motion alone and the updated guidelines.

## Methods and materials

Patients consented to participate in the DELINEATE trial (ISRCTN04483921) were included in a prospective audit, approved by the Trust clinical audit committee. Planning CT scans were acquired (1.5-mm slice thickness) with patients immobilised using the Combi Fix system (Oncology Systems Ltd, UK). Patients were asked to drink 350 ml of water 60 min prior to treatment to maintain a comfortably full bladder throughout treatment. Enemas were prescribed for two days prior to both the planning CT scan and start of treatment, and on each treatment day thereafter.

Volumetric Modulated Arc Therapy (VMAT) plans were created (RaySearch laboratories, Sweden) with flattening filer-free beams. PTV margins were 6 mm in the left (L), right (R), superior (S), inferior (I) and anterior (A) directions and 3 mm posteriorly (P). 36.25 Gy in five fractions was delivered to the prostate, with a concomitant boost of up to 45 Gy to the intraprostatic tumour nodule.

Pre- and post-radiotherapy treatment Cone beam CT (CBCT) images were acquired daily. Pre-treatment images were registered online by two radiographers (plus a clinician on the first fraction). Images were matched using dual registration:initial bony anatomy registration, followed by fiducial registration (XVI Synergy v5.1). Automatic couch correction was performed in three translations with no rotational correction. The time between the start of the pre-treatment CBCT and the start of the post-treatment CBCT was recorded. The time from CBCT acquisition to treatment delivery was recommended to be <2 min.

The post-treatment CBCTs were retrospectively registered as above. Images demonstrating motion greater than the CTV-PTV margins were further examined to determine possible reasons for motion.

The relationship between intra-fraction motion and pre-treatment table correction, overall treatment time and number of VMAT arcs was tested using Spearman’s rank correlation (*ρ*).

Intra-fraction motion margins were calculated using 2.5∑ + 0.7σ,^21^: where ∑ is systematic patient set-up error calculated from the standard deviation (SD) of the mean errors for each patient, and σ is random patient set-up errors calculated from the mean of the SD of each patients’ random errors.^22^

The recently published BIR guidelines^23^ were then followed to calculate margins accounting for all uncertainties in daily online IGRT. Total random and systematic treatment errors were calculated as:

Systematic errors (∑)

$$\sum_{total}^{2}=\sum_{delineation}^{2}+\sum_{fusion}^{2}+\sum_{deformation}^{2}+\sum_{rotation}^{2}+\;\sum_{intra-fraction}^{2}+\sum_{surrogate}^{2}+\sum_{match}^{2}+\sum_{technical\;accuracy}^{2}$$

Random errors (σ)

$$\sigma_{total}^{2}=\sigma_{deformation}^{2}+\sigma_{rotation}^{2}+\sigma_{intra-fraction}^{2}+\sigma_{surrogate}^{2}+\sigma_{match}^{2}+\;\sigma_{technical\;accuracy}^{2}$$

Where:

$$CTV-PTV\;Margin=\alpha \Sigma_{total}+\beta \left[\sqrt{\sigma_{total}^{2}+\sigma_{penumbra}^{2}}-\sigma_{penumbra}\right]$$

$$\sigma_{penumbra}=\frac{d_{D1-D2}}{{CGF}_{1}-{CGF}_{2}}$$

Such that d_D1-D2_ is the distance between suitable isodoses to account for random motion throughout treatment, and CGF_1_ and CGF_2_ are the corresponding cumulative Gaussian function values, D1 was chosen to be 80% as this approximately corresponds to the covering isodose and D2 was chosen to be 60% as an appropriate isodose to cover random errors. d_D1-D2_ was measured and averaged (Supplementary Material 1) for five random patients at the level of the isocentre or centrally within the prostate if the isocentre was excessively offset within the CTV.

Supplementary Material 1.

RL and AP averages were considered similar enough for σ_penumbra_ to be calculated as single values (7.5 mm and 8.1 mm, respectively), SI were calculated separately (superior = 5.1 mm and inferior 2.5 mm).

### α and β values

An α value of 2.5 was chosen for a confidence level of 90% coverage of the target with the prescription isodose, with the assumption that the prostate is a single target.

SBRT plans have a steep dose gradient at the edge of the PTV compared to conventional radiotherapy. A β value of 0.84 has been chosen as recommended for SBRT treatments in which the PTV is covered by 80% of the dose at the centre of the volume.^23^ The presence of the 45 Gy boost volume has not been considered in the selection of the β value as this is usually small enough not to substantially impact the dose gradient at the edge of the prostate PTV.

### Delineation errors

Delineation errors were taken from a range of 5 clinical oncologists contouring the prostate on MRI, using 3 test cases (Appendix 1). The error was calculated as the root mean square of the sum of standard deviations, for each observer’s average distance from a reference contour in each direction^23^ measured centrally within the prostate. The reference contour was created from a simultaneous truth and performance level estimation (STAPLE) of 3 consultant clinical oncologists.

### Rotation errors

Rotational errors were determined using 50 mm prostate diameter (d_x/y/z_=25mm) and 0.5 shape factor (SF_x/y/z_)^23^ such that:

$$\sum_{x}^{2}={\left({\mathrm{d}}_{z}\cdot SF_{y}\cdot \sin\left(\sum_{\theta y}\right)\right)}^{2}+{\left({\mathrm{d}}_{y}\cdot {SF}_{z}\cdot \sin\left(\sum_{\theta z}\right)\right)}^{2}$$

$$\sum_{y}^{2}={\left({\mathrm{d}}_{z}\cdot SF_{x}\cdot \sin\left(\sum_{\theta x}\right)\right)}^{2}+{\left({\mathrm{d}}_{x}\cdot {SF}_{z}\cdot \sin\left(\sum_{\theta z}\right)\right)}^{2}$$

$$\sum_{z}^{2}={\left({\mathrm{d}}_{y}\cdot SF_{x}\cdot \sin\left(\sum_{\theta x}\right)\right)}^{2}+{\left({\mathrm{d}}_{x}\cdot {SF}_{y}\cdot \sin\left(\sum_{\theta y}\right)\right)}^{2}$$

$$\sigma_{x}^{2}={\left({\mathrm{d}}_{z}\cdot SF_{y}\cdot \sin\left(\sigma_{\theta y}\right)\right)}^{2}+{\left({\mathrm{d}}_{y}\cdot {SF}_{z}\cdot \sin\left(\sigma_{\theta z}\right)\right)}^{2}$$

$$\sigma_{y}^{2}={\left({\mathrm{d}}_{z}\cdot SF_{x}\cdot \sin\left(\sigma_{\theta x}\right)\right)}^{2}+{\left({\mathrm{d}}_{x}\cdot {SF}_{z}\cdot \sin\left(\sigma_{\theta z}\right)\right)}^{2}$$

$$\sigma_{z}^{2}={\left({\mathrm{d}}_{y}\cdot SF_{x}\cdot \sin\left(\sigma_{\theta x}\right)\right)}^{2}+{\left({\mathrm{d}}_{x}\cdot {SF}_{y}\cdot \sin\left(\sigma_{\theta y}\right)\right)}^{2}$$

where ∑_Θx/y/z_ and σ _Θx/y/z_ are the systematic and random rotation errors determined from CT-CBCT registration of the fiducials in each plane, using the post treatment CT compared to the reference CT.

### Intra-Fraction errors

Intra-fraction motion was determined as the post treatment couch movement registration, meaning that any residual error in either registration and/or couch movement was included. This will be referred to as intra-fraction motion henceforth.

Since pre- and post-treatment imaging can overestimate intra-fraction motion, the motion was divided by √2 .^23^

### Fusion and surrogate errors

Fusion errors were estimated as zero because as although a fused CT/MRI was used for initial delineation, the contours were reviewed on the planning CT before proceeding.The errors due to fusion should be insignificant compared to (and potentially partially included in) the measured delineation errors. In previous studies using our implantation of markers techniques, no migration of markers was determined,^24^ therefore surrogate error was assumed to be zero.

### Deformation errors

The average deformation through a course of radiotherapy has been defined as <1 mm^25^ and as such systematic deformation is taken to be insignificant due to the short length of a SBRT treatment course. Random deformation was not measured as part of this study.

### Match errors

These are defined for each individual observer *r* by:

$$\sigma_{match}^{2}=\frac{\tau^{2}N_{r}}{N_{r}-1}$$

$$\Sigma_{match}^{2}=T^{2}-\sigma_{match}^{2}$$

With the large number of observers(*n* = 26 radiographers, *n* = 5 clinicians) the random error is approximated by the average variance between observers (τ),the mean variances for each fraction (T) and observer. The resulting error was considered negligible compared to other sources of error, consistent with previous studies using both fiducial markers and online IGRT.^23,26–28^

### Technical accuracy errors

As summing the tolerances of these measurements in quadrature produced errors much larger than those seen in practice, the alternative method of calculating errors by summing the SD of measurements in quadrature was used.^23^ Six months of QA data were analysed for two treatment units for systematic errors, which included MV to kV isocentre and alignmentand table movement (Appendix 1). Systematic MLC errors were calculated as the standard deviation of 6 months of QA data for radiation and light field incidence measurements for one treatment unit (Appendix 1). Random errors were taken from daily QA measurements of the alignment between the MV and kV imaging systems, and optical display indicator (ODI) *vs* a set couch height over 2 weeks for the same two treatment units (Appendix 1). This gives random technical accuracy errors of 0.6 mm RL 0.6 mm SI and 0.9 mm AP. Systematic technical accuracy errors were 0.7 mm RL 0.6 mm SI and 0.4 mm AP.

## Results

97 pairs of pre- and post-treatment CBCT images were retrospectively registered from 20 patients. Three post-treatment images were not acquired, the reason for which was not recorded.

### Average intra-fraction prostate motion by patient

**Figure 1.**
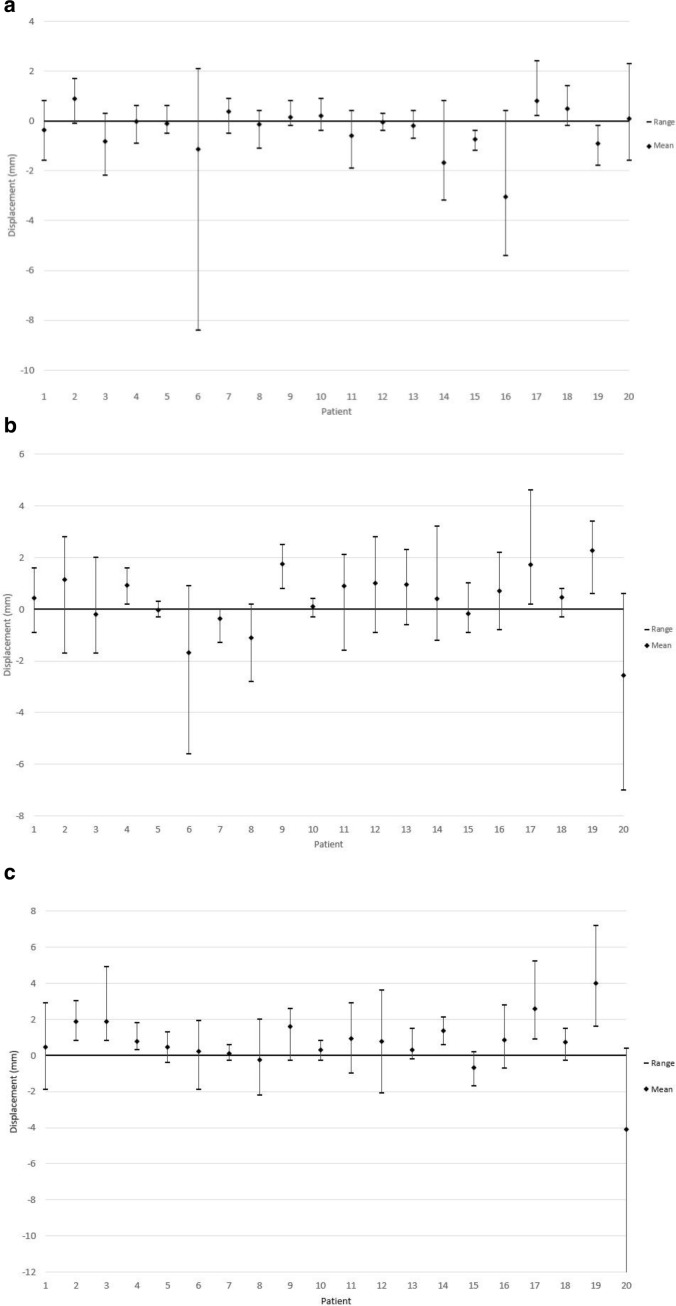
(**a**) Average and maximum displacements in each direction in the RL plane on the post-treatment CBCT compared to the pre-treatment (right positive) (**b**) Average and maximum displacements in each direction in the SI plane on the post-treatment CBCT compared to the pre-treatment (inferior positive) (**c**) Average and maximum displacements in each direction in the AP plane on the post-treatment CBCT compared to the pre-treatment (inferior positive)

Mean (2SD) intra-fraction motions for all patients are 0.4 mm (±3.1 mm), 0.4 mm (±3.4 mm) and 0.7 mm (±4.3 mm) in the RL, SI and AP directions, respectively, where positive denotes right, inferior and posterior (Figure 1).

**Figure 2.**
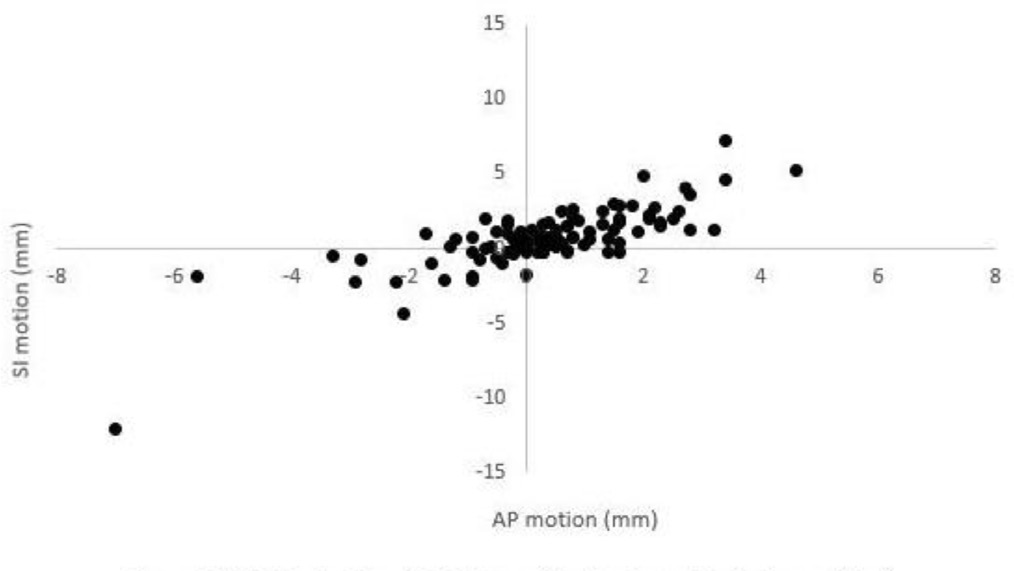
Motion in SA and AP planes (posterior and inferior positive

The majority of intra-fraction motion in the SI plane was in the inferior direction (63%) and in the AP plane was in the posterior direction (70%) (Figure 2) and the two were strongly correlated (*ρ* = 0.72). RL motion was evenly divided.

Most intra-fraction motion in each plane was less than 2 mm (Table 1)

**Table 1.** Intra-fraction prostate motion by direction.

Intra-fraction motion (mm)	Right	Left	Total R/L	Inf	Sup	Total S/I	Post	Ant	Total A/P
0 to 2.0	41	44	85	47	29	76	52	23	75
2.1 to 3.0	4	3	7	10	4	14	10	4	14
3.1 to 4.0	0	1	1	3	1	4	1	0	1
4.1 to 5.0	0	2	2	1	0	1	3	1	4
5.1 to 6.0	0	1	1	0	1	1	1	0	1
≥ 6.1	0	1	1	0	1	1	1	1	2
TOTAL	45 (46%)	52 (54%)	97 (100%)	61 (63%)	36 (37%)	97 (100%)	68 (70%)	29 (30%)	97 (100%)

### Intra-fraction motion

**Figure 3.**
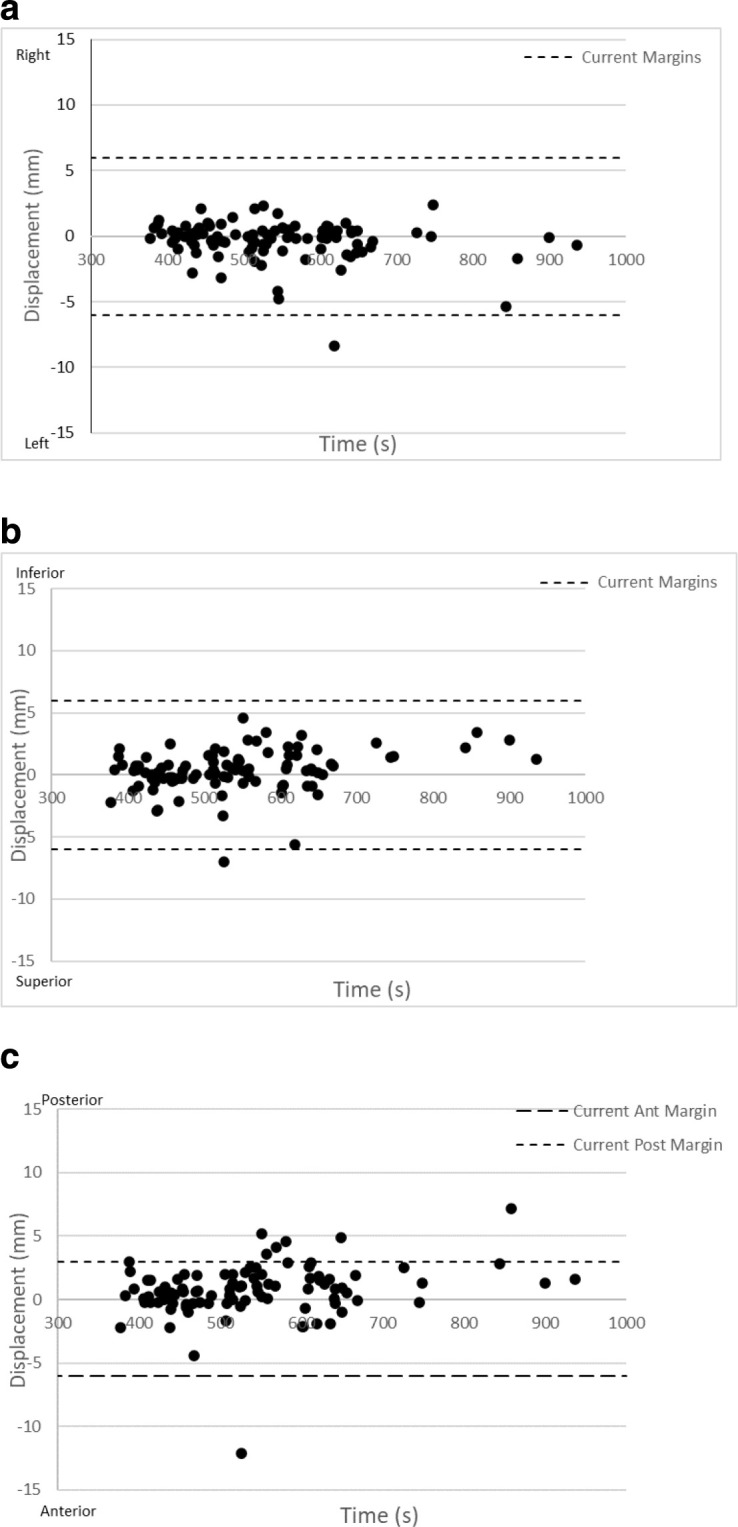
(**a**) Right-left intra-fraction motion vs time with current margins (**b**) Superior-inferior intra-fraction motion vs time with current margins (**c**) Anterior -Posterior intra-fraction motion vs time with current margins

The mean (1SD) time between starting pre- and post-treatment imaging was 9 min (±1 min and 53 sec), the median time was 8 min 46 s. This time includes pre-treatment image acquisition, image registration, couch correction and treatment delivery. The averaged 3D Root Mean Square (RMS) intra-fraction motion (2SD) was 2.5 mm (4.2 mm) for all fractions. There is a weak but significant correlation between the time between pre- and post- imaging and RMS displacement (*r* = −0.288, *p* = 0.004). There was no correlation between time between pre- and post-imaging and absolute motion in lateral direction (*r* = −0.191, *p* = 0.61) and weak but significant corelations in S/I and A/P directions, *r* = −0.311, *p* = 0.002 and *r* = 0.311, *p* = 0.002 s/I and A/P, respectively). There is weak correlation between posterior (significant) and inferior (not significant) intra-fraction motion and time (*r* = 0.35, *ρ* = 0.004=0.31 *p* = 0.018 respectively). Intra-fraction motion greater than current margins was seen in eight fractions, in the superior and anterior directions (*n* = 1), left direction (*n* = 1) and posteriorly (*n* = 6) (Figure 3).

### Causes of motion

Of the eight treatment fractions where intra-fraction motion was greater than the margin, six were evaluated for any underlying causes. Two fractions could not be assessed because the CBCTs were unavailable. Differences in bladder (*n* = 3) and rectum (*n* = 2) volume were identified as contributing causes for motion in four out of six fractions evaluated. Patient movement was seen in another fraction, and it was not possible to identify an underlying cause in the remaining fraction with large superior/anterior shift. There was no correlation between the magnitude of the pre-treatment couch correction and intra-fraction motion (*ρ* = 0.15).

### Intra-fraction motion margins

Systematic intra-fraction motion errors were ≤1 mm in all directions and random errors were larger in the AP direction (Table 2). Rotational errors resulted in margins < 2 mm (Table 3). The margins required to compensate only for intra-fraction motion alone were ≤3.5 mm. 92% of fractions had motion within the margins specified (Table 4). Only 3% of fractions would have required greater than 6 mm margins in any direction. However, 6% (*n* = 6) of fractions had motion greater than the posterior margin.

**Table 2.** Population random and systematic intra-fraction motion errors with corresponding margins

	RL (mm)	SI (mm)	AP (mm)
∑	0.9	1.0	1.0
σ	1.0	1.2	1.4
Current Margins	6	6	6 Ant/3 Post
Calculated intra-fraction motion margins^26^	3.0	3.3	3.5

### Rotational motion margins

**Table 3.** Population random and systematic rotational errors with corresponding equivalent translational values.

	X	Y	Z
∑_θ_	3.5**°**	1.3**°**	1.2**°**
σ_θ_	2.9**°**	1.3**°**	0.9**°**
∑	0.39	0.81	0.81
σ	0.34	0.66	0.69

### Margin calculation

**Table 4.** Population random and systematic margin calculations, calculated using updated guidelines

Systematic						
	Right (mm)	Left (mm)	Superior (mm)	Inferior (mm)	Anterior (mm)	Posterior (mm)
Delineation	0.9	0.8	0.7	2.4	1.3	0.6
Rotational	0.4		0.8		0.8	
Intra-fraction	0.7		0.7		0.7	
Technical Accuracy	0.7		0.6		0.4	
∑_total_	1.4	1.3	1.4	2.7	1.7	1.3
Systematic margin (α = 2.5)	3.5	3.3	3.5	6.7	4.3	3.2
Random						
Rotational	0.3		0.7		0.7	
Intra-fraction	0.7		0.8		1.0	
Technical Accuracy	0.6		0.6		0.9	
σ_total_	1.0		1.2	1.2	1.5	
σ­_penumbra_	7.5		5.1	2.5	8.1	
Random margin(β = 0.84)	<0.1		0.2	0.3	0.1	
Total Margins	3.5	3.3	3.7	7.0	4.4	3.3

## Discussion

### Intra-fraction motion

Mean intra-fraction motion (2SD) is 0.4 mm (±3.1 mm), 0.4 mm (±3.4 mm) and 0.7 mm (±4.3 mm) in the SI, RL and AP directions, respectively, where positive value denotes the right, inferior and posterior displacement. Compared to studies^29–32^ using similar methods of measuring intra-fraction motion,the motion in RL direction is greater, but comparable AP and SI. The greater RL motion is due to two patients (Figures 1–3). The RMS intra-fraction motion within the treatment time was comparable to a similar study,^33^ which also found that treatment times < 5 min reduced intra-fraction motion. The longer treatment times associated with SBRT delivery have been shown to increase intra-fraction motion^34,35^ so any reduction in overall time could be beneficial. However, there was only weak correlation demonstrated between both posterior and inferior motion with time between pre- and post- imaging within our range of times (6–15 min range 9 min average).

### Causes of motion

Intra-fraction motion was predominantly in the inferior and posterior direction, probably due to internal organ motion rather than patient motion. Prostate motion in the inferior and posterior direction has been found to be related to bladder filling.^36^ Although unable to measure bladder volume because of the restricted field of view on the CBCT, it was identified that differences in bladder volume between CT planning and treatment were present. Treating with an empty bladder may provide a more reproducible and reliable method of ensuring consistency between treatments and reduced intra-fraction motion^37^ but potentially at the expense of increased total bladder dose.

Our current treatment protocols recommend an empty rectum at pre-treatment, which has been shown to result in less intra-fraction motion of the prostate.^38^ However, non-effectiveness of the enema can cause intra-fraction motion with no clear intervention available.^39^ In our study large rectal diameter through gas or faeces was noted anecdotally on pre-treatment CBCT on several occasions; in these instances, treatment was delayed allowing for rectum emptying. Further measures to improve consistency of rectal volumes may be warranted.^38,40^

There was no discernible relationship between the magnitude of intra-fraction motion and inter-fraction displacements.

### Margins for prostate SBRT

The current margins (6 mm isotropic except 3 mm posterior) account for 92% of intra-fraction motion. However, current clinical margins are intended to account for all sources of error not only intra-fraction motion.Using the recently published BIR margin guidelines,^23^ resulted in margins of between 3.3 and 7.0 mm (Table 4); suggesting current margins, except in the inferior directions, are adequate and indeed could be decreased laterally which is consistent with other studies.^41^ There may also be other factors at play, for example incidental dose outside the prostate has been related to reduced treatment failure.^42^ The high doses delivered in SBRT maximise this effect. Patient outcome in current trials should be examined carefully prior to any change in practice. The impact of delineation errors remains the largest contribution to the margin. If the potential outlier (doctor 1, case 2) is excluded as suggested,^23^ the resultant inferior margin would be reduced to 5.8 mm (from 7 mm). The slight discrepancy between left-right margins is due to rounded values compounding.

Intra-fraction motion margins determined in this study are close to those in other similarly calculated hypo-fractionated or SBRT studies,^43,44^ where the authors have also highlighted difficulties in delineation at the apex of the prostate.^43^ There was less variation superiorly (<1 mm away from the reference contour for 13 of 15 contours), compared with other directions, particularly the inferior where the greatest variation in contouring is seen (Appendix 1), possibly due to the bladder prostate interface being more easily identified than the apex. Contouring guidance may reduce the outliers.

A similar study calculating margins with a larger patient population (*n* = 205) had smaller systematic errors (0.05 cm RL, 0.07 cm SI and 0.09 cm AP) but similar random errors (0.07 cm RL, 0.13 cm SI and 0.14 cm AP)^45^ The greatest motion was reported to occur between initial imaging and delivery of the first treatment beam, indicating that a reduction in the time taken for image verification, could decrease intra-fraction motion.

### Penumbral values and random error margins

The random errors margin componentis small compared to the systematic errors component due to the choice of isodoses 80%/60% for penumbral coverage of random motion resulting in a $\sigma_{penumbra}$ value much greater than $\sigma_{total}$ . Even with a relatively larger difference in the distance between these isodoses (as in the case for the SI directions) as $\sigma_{penumbra}$ is still much greater than $\sigma_{total}$ , this part of the equation becomes almost in consequential compared to the systematic errors’ component. Including a 20% variation in any of the measurements for random errors and a compounding measurement error in the distances between isodoses, results in a change to the margin of <0.1 mm. In the context of SBRT, random errors having a lesser impact on margins is expected, as there is a chance of random errors causing either an increase or decrease in dose to the periphery of the tumour due to the small distance and relative intensity between dose levels at the edge of the PTV. Therefore, systematic errors which will have an impact on each fraction in the entire treatment course will be of greater importance in SBRT compared to random errors which will potentially only impact on a single treatment.

## Recommendations/Limitations

Deformation was taken to be 0 because some of which would have been included in our intra-fraction and rotational margins, however some residual will remain and could be determined.

Delineation errors were measured from an MRI only study and will therefore be smaller than found on CT or fused MR/CT imaging.^41^ The errors from CT/MR fused images were not measured as part of this audit and should be calculated using the suggested method^23^ to evaluate their impact on margins. These errors will be systematic and therefore potentially have a larger impact on overall margin size compared to other sources of error.

Intra-fraction and rotational components were calculated in each plane, whereas more motion (both magnitude and number of fractions) is present in the inferior and posterior directions. AP and SI may benefit from being calculated separately as this could potentially impact on margins.

The calculations performed assumed match error was negligible, mitigated by many different observers performing the matching online (*n* = 31). However, this assumption has not been measured. A negligible match error is also reliant on a fiducial match being a reliable surrogate for prostate position. Within the context of prostate SBRT, the rapid isodose fall off could easily result in geometric miss if the surrogate is not representative, for example if the fiducial markers are not well spread out within the prostate. Both inter- and intra-observer variation under the conditions of fiducial markers and online IGRT should be measured to assess the impact on margins.

Although the match was based on fiducial alignment, the benefit of utilising CBCT imaging is that miss-alignment or misidentification of fiducials can be visualised, as can target deformation and OAR changes. Soft tissue agreement is reviewed before applying isocentre shifts, reducing the risk of fiducial surrogate error impacting match accuracy. Poor fiducial match accuracy was only observed for one patient (patient 20); this was identified before treatment delivery at fraction one and soft-tissue alignment implemented. We therefore consider any surrogate match error to be very small.

## Conclusion

Current planning margins of 6 mm isotropic, 3 mm posterior for SBRT prostate treatment adequately account for 92% of intra-fraction motion. Intra-fraction motion margins of 3 mm RL, 3.3 mm SI and 3.6 mm would cover 88% of intra-fraction motion. Updated guidelines suggest that margins could be decreased from 6mm in the left/right directions, however, these margins also account for other errors involved in the treatment process. Increasing the inferior margins is suggested, but is predominately due to delineation errors. Since current practice is producing favourable results, the relationship between margins, outcome and toxicity must be closely evaluated prior to changing practice.
