# Supplementary material for: Prostate stereotactic body radiotherapy: quantifying intra-fraction motion and calculating margins using the new BIR geometric uncertainties in daily online IGRT recommendations
Source: Br J Radiol. 2023 Apr 22;96(1146):20220852. doi: 10.1259/bjr.20220852 (PMC10230383; doi:10.1259/bjr.20220852)
Supplement: Supplementary Material 1. [file bjr.20220852.suppl-01.docx]

**Appendix 1.**

**Distance between isodose lines for penumbral values**

| Patient | R (mm) | L (mm) | S (mm) | I (mm) | A (mm) | P (mm) |
| --- | --- | --- | --- | --- | --- | --- |
| 7 | 3.6 | 2.2 | 3.3 | 1.0 | 3.6 | 3.3 |
| 10 | 6.2 | 7.0 | 4.6 | 1.7 | 6.0 | 7.0 |
| 19 | 3.7 | 4.0 | 3.0 | 1.8 | 4.4 | 5.2 |
| 4 | 3.5 | 3.0 | 1.1 | 1.1 | 3.6 | 4.2 |
| 16 | 5.2 | 5.1 | 3.2 | 1.8 | 5.2 | 5.3 |
| Average | 4.44 | 4.26 | 3.04 | 1.48 | 4.56 | 5.00 |
| σ­_penumbra_ | 7.5 | | 5.1 | 2.5 | 8.1 | |
| Distance between 80% and 60% isodose lines for 5 random patients from sample and resulting random penumbral errors. | | | | | | |

**Technical accuracy errors data.**

|  | Unit 1 | | | | | | Unit 2 | | | | | |  | |
| --- | --- | --- | --- | --- | --- | --- | --- | --- | --- | --- | --- | --- | --- | --- |
| Laser to MV (mm) | Month 1 | Month 2 | Month 3 | Month 4 | Month 5 | Month 6 | Month 1 | Month 2 | Month 3 | Month 4 | Month 5 | Month 6 | **SD** |  |
| RL | 0.16 | 0.17 | -0.46 | -0.50 | -0.70 | -0.59 | 0.03 | 0.32 | -0.13 | -0.30 | -0.21 | -0.35 | **0.31** |  |
| SI | 0.61 | 0.23 | -0.29 | -0.77 | -0.39 | -0.11 | -0.27 | 0.08 | -0.43 | -0.31 | 0.01 | -0.07 | **0.34** |  |
| AP | 0.04 | 0.23 | 0.29 | 0.37 | -0.01 | -0.01 | -0.25 | -0.10 | -0.08 | 0.05 | -0.37 | -0.19 | **0.21** |  |
|  |  |  |  |  |  |  |  |  |  |  |  |  |  |  |
| MV to kV (mm) | Month 1 | Month 2 | Month 3 | Month 4 | Month 5 | Month 6 | Month 1 | Month 2 | Month 3 | Month 4 | Month 5 | Month 6 | **SD** |  |
| RL | 0.1 | 0.3 | -0.1 | 0.1 | -0.5 | 0.1 | 0.1 | 0.1 | -0.3 | 0.1 | -0.1 | 0.1 | **0.21** |  |
| SI | 0.1 | -0.1 | 0.0 | 0.2 | -0.2 | -0.2 | -0.2 | -0.3 | 0.0 | -0.1 | -0.2 | 0.0 | **0.14** |  |
| AP | 0.2 | 0.4 | 0.2 | 0.0 | 0.0 | 0.0 | 0.1 | -0.4 | 0.0 | 0.1 | 0.0 | -0.1 | **0.18** |  |
|  |  |  |  |  |  |  |  |  |  |  |  |  |  |  |
| Couch move (mm) | Month 1 | Month 2 | Month 3 | Month 4 | Month 5 | Month 6 | Month 1 | Month 2 | Month 3 | Month 4 | Month 5 | Month 6 | **SD** |  |
| RL | 0.5 | 0.5 | -1.0 | -1.0 | -0.5 | -0.5 | 0.5 | 1.0 | -0.5 | -0.5 | 0.0 | -0.5 | **0.62** |  |
| SI | 0.0 | 0.0 | -1.0 | -1.0 | 0.0 | -0.5 | 0.0 | 0.0 | 0.0 | 0.0 | 0.5 | 0.0 | **0.42** |  |
| AP | 0.5 | 0.0 | -0.5 | 0.0 | 0.5 | 0.0 | 0.0 | 0.5 | 0.5 | 0.5 | 0.5 | 0.0 | **0.32** |  |
| Systematic error QA data for 6 months for 2 treatment units. | | | | | | | | | | | | | | |

|  | Unit 1 | | | | | Unit 2 | | | | |
| --- | --- | --- | --- | --- | --- | --- | --- | --- | --- | --- |
|  | Daily SBRT MV – kV Check (mm) | | | | Couch Height – ODI (mm) | Daily SBRT MV – kV Check (mm) | | | | Couch Height – ODI (mm) |
| **Day** | **LR** | **SI** | **SI** | **AP** | **AP** | **LR** | **SI** | **SI** | **AP** | **AP** |
| 1 | 0.5 | 0 | 1.3 | 0 | 2 | 1 | 0.8 | 0 | 0.5 | 0 |
| 2 | 0.8 | 0.5 | 0.3 | 0 | 2 | 2 | -0.8 | 1.2 | -0.5 | 0 |
| 3 | 0.5 | 0.5 | 0.3 | 0.5 | 1 | -0.3 | 0.5 | 0.5 | -1.3 | 1 |
| 4 | 1 | 0.5 | 0.5 | 0.3 | 1 | 0.8 | 0.5 | 0.5 | 0.8 | 1 |
| 5 | 0 | 0.3 | 1 | 0 | 2 | 0.3 | 0.3 | -0.3 | 1.3 | 1 |
| 6 | 0.5 | 0 | 0.8 | 0.3 | 1 | -0.8 | 0.3 | 0.8 | 1 | 1 |
| 7 | 0.5 | 0.5 | 0.3 | 0.3 | 1 | 0.3 | 0.5 | 0.3 | 0.3 | 1 |
| 8 | 0.5 | 0.8 | 0.3 | 0.8 | 1 | 0 | 0.8 | 0.8 | 0.8 | 1 |
| 9 | 1.3 | 0 | 0 | -0.3 | 2 | 0.8 | -0.5 | 0 | 0 | 0 |
| 10 | 0 | -0.5 | 0.5 | -0.3 | 1 | 1.3 | 1.3 | 0.5 | 1.5 | 1 |
| **SD** | 0.38 | 0.36 | 0.37 | 0.33 | 0.49 | 0.60 | 0.34 | 0.18 | 0.81 | 0.46 |
| Random error daily QA checks for 2 weeks for 2 treatment units. | | | | | | | | | | |

**Prostate Contouring Data.**

| **Doctor** | **Anterior** | **Posterior** | **Right** | **Left** | **Superior** | **Inferior** |
| --- | --- | --- | --- | --- | --- | --- |
| 1 | 2.6 | -0.6 | 1.6 | 0.5 | 0 | 0 |
| 2 | -0.7 | 0.3 | 0.8 | -0.6 | 2 | 2.6 |
| 3 | -1.4 | -0.9 | 0 | -0.8 | 5 | 0 |
| 4 | 0.7 | 0.4 | 0 | 0 | 0 | 0 |
| 5 | -1.4 | 0.3 | -0.7 | -0.7 | 0 | -2.4 |
| 1 | -2.1 | -0.5 | 0.6 | 0.8 | 0 | -5.2 |
| 2 | 0 | -0.5 | 1.1 | 0 | 0 | 0 |
| 3 | -0.2 | -1 | -0.3 | 0 | 0 | 0 |
| 4 | 0.5 | -1 | -0.3 | -0.6 | 0 | 0 |
| 5 | 0.8 | -1.4 | -1.2 | -1.2 | 0 | 2.4 |
| 1 | 1.8 | -0.2 | 0.9 | -0.4 | 0 | 0 |
| 2 | -0.5 | -1.3 | 1.3 | -2.6 | 0 | -2.8 |
| 3 | -0.8 | 0 | 1.6 | -1.9 | 0 | 2.6 |
| 4 | -1 | 0 | 2.3 | 0 | 0 | 4 |
| 5 | -0.5 | 0.8 | 0 | 0 | 0 | 0 |
| **Combined**  **Systematic Error** | **1.3** | **0.6** | **0.9** | **0.8** | **0.7** | **2.4** |
| 5 doctors contouring distance from a reference contour for 3 patient cases. All distances in mm. | | | | | | |
